# Supplementary material for: Tailoring CO2/CH4 Separation Performance of Mixed Matrix Membranes by Using ZIF-8 Particles Functionalized with Different Amine Groups
Source: Polymers (Basel). 2019 Dec 9;11(12):2042. doi: 10.3390/polym11122042 (PMC6960569; doi:10.3390/polym11122042)
Supplement: Supplementary file 1 [file polymers-11-02042-s001.pdf]

Supplementary File

Tailoring CO_2/CH_4 separation performance of mixed matrix membranes by using ZIF-8 particles functionalized with different amine groups

Nadia Hartini Suhaimi[1,2], Yin Fong Yeong[1,2, *], Christine Wei Mann Ch'ng[1,2] and Norwahyu Jusoh[1,3]

[1] Chemical Engineering Department, Universiti Teknologi PETRONAS, 32610 Seri Iskandar, Perak.
[2] CO_2 Research Centre (CO2RES), R&D Building, Universiti Teknologi PETRONAS, 32610 Seri Iskandar, Perak.
[3] Centre for Contaminant Control & Utilization (CenCoU), Chemical Engineering Department, Universiti Teknologi PETRONAS, 32610 Seri Iskandar, Perak.

* Correspondence: yinfong.yeong@utp.edu.my ; Tel +605-3687564

Contents

1. Supplementary Table S1, Figure S1 and Figure S2

Table S1. *Elemental analysis data of ZIF-8 and amine-functionalized ZIF-8 particles.*

Sample	N (wt%)	C (wt%)	H (wt%)
ZIF-8	20.75	35.97	3.78
APTMS-ZIF-8	22.89	40.34	4.41
AAPTMS-ZIF-8	22.58	40.53	4.46
AEPTMS-ZIF-8	22.10	40.65	4.66

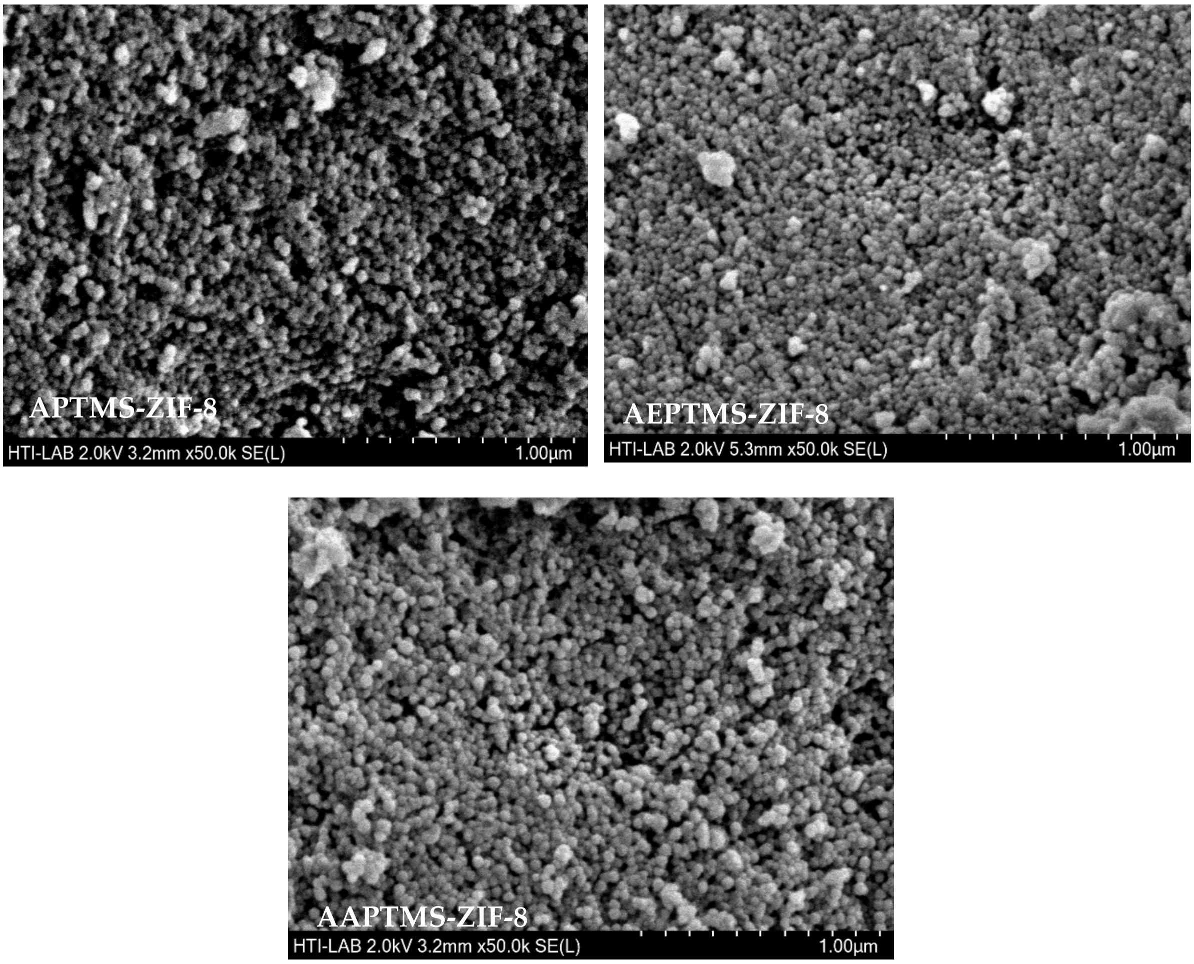

Figure S1. *Field emission scanning electron microscope (FESEM) images of amine-functionalized ZIF-8 particles.*

a) Spectrum: Point

Element	AN	Series	norm. C [wt.%]	Atom. C [at.%]
Carbon	6	K-series	55.44	63.55
Fluorine	9	K-series	18.17	13.17
Oxygen	8	K-series	18.13	15.60
Nitrogen	7	K-series	7.70	7.57
Zinc	30	K-series	0.56	0.12
		Total:	100.00	100.00

b) Spectrum: Point

Element	AN	Series	norm. C [wt.%]	Atom. C [at.%]
Carbon	6	K-series	58.25	66.18
Oxygen	8	K-series	18.86	16.09
Fluorine	9	K-series	16.33	11.73
Nitrogen	7	K-series	6.05	5.90
Zinc	30	K-series	0.50	0.11
		Total:	100.00	100.00

c) Spectrum: Point

Element	AN	Series	norm. C [wt.%]	Atom. C [at.%]
Carbon	6	K-series	54.02	62.15
Fluorine	9	K-series	18.67	13.58
Oxygen	8	K-series	18.57	16.04
Nitrogen	7	K-series	8.23	8.12
Zinc	30	K-series	0.52	0.11
		Total:	100.00	100.00

d) Spectrum: Point

Element	AN	Series	norm. C [wt.%]	Atom. C [at.%]
Carbon	6	K-series	50.74	59.36
Oxygen	8	K-series	24.78	21.76
Fluorine	9	K-series	15.17	11.22
Nitrogen	7	K-series	7.19	7.21
Zinc	30	K-series	2.12	0.45
		Total:	100.00	100.00

e) Spectrum: Point

Element	AN	Series	norm. C [wt.%]	Atom. C [at.%]
Carbon	6	K-series	58.75	67.31
Fluorine	9	K-series	17.75	12.86
Oxygen	8	K-series	13.05	11.22
Nitrogen	7	K-series	8.29	8.15
Zinc	30	K-series	2.15	0.45
		Total:	100.00	100.00

f) Spectrum: Point

Element	AN	Series	norm. C [wt.%]	Atom. C [at.%]
Carbon	6	K-series	53.52	62.38
Fluorine	9	K-series	18.75	13.82
Oxygen	8	K-series	18.35	16.06
Nitrogen	7	K-series	7.30	7.30
Zinc	30	K-series	2.08	0.45
		Total:	100.00	100.00

Figure S2. *The atomic (%) of Zn elements of the membrane (a) M1; (b) M2; (c) M3; (d) M4; (e) M5 and (f) M6.*
